# A brief unstructured literature review on the history of paraphilias

**DOI:** 10.1038/s41443-024-00835-4

**Published:** 2024-02-13

**Authors:** Safiye Tozdan

**Affiliations:** Institute for Sex Research, Sexual Medicine and Forensic Psychiatry, University Medical Center Hamburg-Eppendorf, Hamburg, Germany

**Keywords:** Medical research, Quality of life

## Abstract

This unstructured review is based on a comprehensive literature search leading to a variety of selected studies that summarize the historical development of paraphilias. Firstly, paraphilias in ancient times are discussed. Secondly, the development of paraphilia diagnoses, including current critical aspects, is outlined. Finally, a short description of the development of treatment approaches for individuals with paraphilic disorders and those who commit sexual offenses, including medical and psychotherapeutic approaches as well as online intervention programs, is presented. The destigmatization of people with deviant sexual interest is deemed necessary. However, it is also recommended to always strive for a balance between protecting paraphilic individuals’ rights and protecting vulnerable groups to whom paraphilic people can pose a danger.

## Introduction

The term “paraphilias” consists of two Greek words: 1. “para” which is translated as various meanings like besides, beyond, abnormal, defective, irregular, or altered; and 2. “philias” which means love, friendship, brotherly love or affection [1]. Thus, commonly understood, paraphilia describes “love beyond the usual to abnormal love or sexuality.” [2]. Since the late 19th century, the construct of paraphilias and paraphilia diagnoses have always been discussed by experts in the field. Simultaneously, the treatment of paraphilic disorders has evolved over time – from barbaric methods to ethically justifiable medicinal and psychotherapeutic methods. To understand paraphilias in today’s time, a look back in history discovering the developments regarding paraphilias in the past is deemed necessary [3]. Therefore, the following unstructured literature review includes selected studies on the history of paraphilias describing paraphilias in ancient times, outlining the development of paraphilia diagnoses including current critical aspects mentioned by experts, and presenting the evolvement of treatment approaches for individuals with paraphilic disorders and individuals who commit sexual offenses. The article ends with a conclusion.

## Methods

This article is no systematic review and does not claim completeness. The author chose a comprehensive literature search leading to a variety of selected studies that summarize the historical development of paraphilias in research and clinical practice. The selected studies mainly included reviews which led the author to corresponding articles on the topic of paraphilias that were also taken into account. In the selection of all studies on the history of paraphilias, the author aimed to present the development of the construct of paraphilias as well as the development of diagnoses and treatment approaches. Search terms included “paraphilias”, “paraphilic disorder”, “history”, “diagnoses”, “DSM”, “ICD”, “sexual offenses”, and related terms. All literature searches were performed in suitable databases devoted to peer-reviewed literature in the behavioral sciences and mental health (PsychInfo, PubMed). Additional references were used where it seemed useful or necessary (e.g., Google Scholar).

## Results and discussion

### Paraphilias in ancient times

Although it is considered difficult to ascertain whether paraphilias existed before the Renaissance [4], paraphilic behavior might be as old as civilized humanity. For example, paraphilic behavior has even been described in the Bible [5]. Exhibitionistic behavior has existed since the description of Adam and Eve. Since civility, undressing in public has been viewed as pathological and often associated with madness [6]. Moreover, transvestism was prohibited in the Bible: “The woman shall not wear that which pertaineth unto a man, neither shall a man put on a woman’s garment” [7]. Sadistic and masochistic behaviors have been described in the Kamasutra (e.g., consensual erotic slapping) which was probably written between 200 and 300 A.D. [3]. In ancient Greece, it was common for boys to be sexually exploited by adult men [8]. These abusive acts were disguised as mentoring relationships between teachers and students. Later, during the 4th century B.C., Plato advocated that sexual acts with children be punished [3].

Interestingly, some paraphilias got their names from individuals who suffered from them in the past. The French nobleman Marquis de Sade (1740–1814) was aroused by violent fantasies. He became known for his violent pornographic novels, most of which he wrote during decades of stay in prisons and mental asylums. The paraphilia in which a person experiences pleasure in violating the physical or psychological integrity of another living being was called “sadism” later. Another example is the Baron Leopold von Sacher-Masoch (1835–1895) who has given his name to the paraphilia “masochism” during his lifetime. The author became famous for his imagination and art of esthetically formulating instinctual desires for pain and submission [9]. In 1905, Freud claimed that sadism and masochism often occur together and introduced the term “sadomasochism”. However, both paraphilias are still seen as separate phenomena [10]. Another paraphilic behavior that was described in the 19th century is voyeurism. Even back then, people paid well to look through “peepholes” to watch others having sexual intercourse, especially in Parisian brothels [11]. Furthermore, frotteuristic behavior (i.e., touching and rubbing against non-consenting individuals to gain sexual pleasure) was mentioned in 1887 and considered to be associated with a mental disorder [3].

Physicians of the 19th century began to have a very powerful position in society, comparable to clergy. For example, they were often called by police for guidance on how to deal with people who committed sexual offense. In addition, psychiatry became a medical discipline and illness was no longer limited to the body but also to the mind. In the further course, a definition of insanity was developed that included disorders of the will and the emotions. Finally, this led to a psychiatric account for sexually deviant interests [12]. In that time, paraphilias were called “perversions”; a term dated back to the Middle Ages and the Renaissance when “any act that violated the laws of God was considered a perversion” [12]. With the publication of Krafft-Ebing’s “Psychopathia Sexualis”, paraphilias were proposed to be considered as medical conditions [9]. Since then, researchers and clinicians have debated paraphilias for a long time (and will probably continue to do so in the future). Numerous changes have taken place regarding the definitions and diagnoses of paraphilias [2]. In the following, a brief description of how paraphilia diagnoses arise is presented. In addition, several points of criticism mentioned by researchers and clinicians are delineated.

### Development of diagnoses for paraphilias

In 1948, the sixth version of the “International Statistical Classification of Diseases and Related Health Problems” (ICD-6) contained a large section for mental disorders for the first time which also included paraphilias. Subsumed under “pathogenic personality”, they were called “sexual deviation” [13]. They were described as non-reproductive sexual behaviors and included exhibitionism, fetishism, homosexuality, pathologic sexuality, and sadism. This classification was carried forward in the ICD-7 [14] and in ICD-8 [15]. The term “disorders of sexual preferences” was introduced by the ICD-9 [16] and subsequently classified as a subcategory of “disorders of adult personality and behavior” in the ICD-10 [17]. Similar developments emerged regarding the “Diagnostic and Statistical Manual of Mental Disorders” (DSM). The first [18] and second versions of the DSM [19] included paraphilias as “sexual deviation” under the category “sociopathic personality disorders”. The manuals specified: ”Individuals to be placed in this category are ill primarily in terms of society and of conformity with the prevailing cultural milieu, and not only in terms of personal discomfort and relations with other individuals.” [19]. Diagnoses to be made were homosexuality, transvestism, pedophilia, fetishism, and sexual sadism (including rape, sexual assault, and sexual mutilation) [2]. However, no detailed descriptions of specific disorders were made [20]. The DSM-III in 1980 included the term “paraphilias” for the first time which were subsumed under “psychosexual disorders” [21]. Back then, paraphilias were described as atypical sexual arousal to objects, situations, or nonconsenting individuals [22]. In the context of DSM-IV [23], a duration criterion was added for paraphilias that required the symptoms to be present for more than 6 months, causing clinically significant distress or impairment in social, occupational, or other important areas of functioning. Furthermore, the DSM-IV was driven by the attempt to be more based on research evidence. Thus, work groups consisting of experts in the field were set up to support this process [20].

Over time and due to societal development and the efforts of several organizations built by gay and lesbian communities, paraphilias became the subject of critical debates. The accusation addressed the fact that some paraphilic behaviors can take place without any clinical distress or threat to others. For example, individuals can engage in consensual sado-masochistic activities [2]. By pathologizing such behavior, some people even were put on trial and found guilty of assault in the past [24]. Therefore, researchers and clinicians began to agree that some paraphilia diagnoses in the DSM and ICD are outdated and highly stigmatizing, such as fetishism and sadomasochism (e.g., [25]). Thus, the latest diagnostic manuals (ICD-11: [26]; DSM-5: [27]) distinguish between paraphilias as paraphilic disorders taking into account that paraphilic interests do not necessarily indicate clinical distress and/or a threat to others [28]. Therefore, the diagnoses of fetishism, fetishistic transvestism, and sadomasochism have been removed from the ICD-11. Instead, the new paraphilic disorder “coercive sexual sadism disorder” was added to differentiate between the disorder and consensual sadomasochistic behaviors that do not include harm or risk. A paraphilic disorder can only be diagnosed if the individual has acted on these thoughts, fantasies or urges or is markedly distressed by them [26]. Similarly, in the DSM-5, the term “paraphilia” is defined as any intense and persistent atypical sexual interest. “Paraphilic disorder” is in addition related to personal distress, personal harm, social impairment, and/or risk of harm to others. Moreover, the personal distress about the paraphilic interest should not be merely distress resulting from society’s disapproval [27]. Hence, the DSM-5 takes into account that people can engage in atypical sexual behaviors without being defined as having a mental disorder [20]. The paraphilic disorders included in the DSM-5 and the ICD-11, their description, and diagnostic criteria are presented in Table 1. For prevalence rates of the different paraphilic disorders, we refer the reader for example to Beech et al. [20]. Although diagnostic criteria for paraphilias have undergone continuous changes in the past taking account proposals by numerous experts in the field, some clinicians and researchers still criticize current developments. Some of them are described in the following.

**Table 1:** Short description of the DSM-5 and ICD-11 paraphilic disorders and diagnostic criteria.

Paraphilic Disorder	DSM 5 diagnostic criteria	ICD-11 diagnostic criteria
Voyeuristic Disorder	A) Over a period of at least 6 months, recurrent and intense sexual arousal from observing an unsuspecting person who is naked, in the process of disrobing, or engaging in sexual activity, as manifested by fantasies, urges, or behaviors. B) The individual has acted on these sexual urges with a nonconsenting person, or the sexual urges or fantasies cause clinically significant distress or impairment in social, occupational, or other important areas of functioning. C) The individual experiencing the arousal and/or acting on the urges is at least 18 years of age.	A) A sustained, focused and intense pattern of sexual arousal—as manifested by persistent sexual thoughts, fantasies, urges, or behaviors—that involves stimuli such as observing an unsuspecting person who is naked, in the process of disrobing, or engaging in sexual activity. B) The individual must have acted on these thoughts, fantasies or urges or be markedly distressed by them. *Additional Features:* The act of observing is for the purpose of achieving sexual excitement and does not necessarily involve an attempt to initiate sexual activity with the person being observed. Orgasm by masturbation may occur during the voyeuristic activity or later in response to memories of what the individual has seen. More recently, so-called ‘video voyeurs’ have been described who use video equipment to record individuals in public or private places where there is an expectation of privacy.
Exhibitionistic disorder	A) Over a period of at least 6 months, recurrent and intense sexual arousal from the exposure of one’s genitals to an unsuspecting person, as manifested by fantasies, urges, or behaviors. B) The individual has acted on these sexual urges with a nonconsenting person, or the sexual urges or fantasies cause clinically significant distress or impairment in social, occupational, or other important areas of functioning.	A) A sustained, focused and intense pattern of sexual arousal—as manifested by persistent sexual thoughts, fantasies, urges, or behaviors—that involves exposing one’s genitals to an unsuspecting person in public places, usually without inviting or intending closer contact. B) The individual must have acted on these thoughts, fantasies or urges or be markedly distressed by them.
Frotteuristic Disorder	A) Over a period of at least 6 months, recurrent and intense sexual arousal from touching or rubbing against a nonconsenting person, as manifested by fantasies, urges, or behaviors. B) The individual has acted on these sexual urges with a nonconsenting person, or the sexual urges or fantasies cause clinically significant distress or impairment in social, occupational, or other important areas of functioning.	A) A sustained, focused and intense pattern of sexual arousal—as manifested by persistent sexual thoughts, fantasies, urges, or behaviors—that involves touching or rubbing against a non-consenting person in public places. B) The individual must have acted on these thoughts, fantasies or urges or be markedly distressed by them.
Sexual Masochism Disorder	A) Over a period of at least 6 months, recurrent and intense sexual arousal from the act of being humiliated, beaten, bound, or otherwise made to suffer, as manifested by fantasies, urges, or behaviors. B) The fantasies, sexual urges, or behaviors cause clinically significant distress or impairment in social, occupational, or other important areas of functioning.	
DSM.5: Sexual Sadism Disorder ICD-11: Coercive Sexual Sadism Disorder	A) Over a period of at least 6 months, recurrent and intense sexual arousal from the physical or psychological suffering of another person, as manifested by fantasies, urges, or behaviors. B) The individual has acted on these sexual urges with a nonconsenting person, or the sexual urges or fantasies cause clinically significant distress or impairment in social, occupational, or other important areas of functioning.	A) A sustained, focused and intense pattern of sexual arousal—as manifested by persistent sexual thoughts, fantasies, urges or behaviors—that involves the infliction of physical or psychological suffering on a non-consenting person B) The individual must have acted on these thoughts, fantasies or urges or be markedly distressed by them.
Pedophilic Disorder	A) Over a period of at least 6 months, recurrent, intense sexually arousing fantasies, sexual urges, or behaviors involving sexual activity with a prepubescent child or children (generally age 13 years or younger). B) The individual has acted on these sexual urges, or the sexual urges or fantasies cause marked distress or interpersonal difficulty. C) The individual is at least age 16 years and at least 5 years older than the child or children in Criterion A.	A) A sustained, focused, and intense pattern of sexual arousal—as manifested by persistent sexual thoughts, fantasies, urges, or behaviors—involving pre-pubertal children. B) The individual has acted on these thoughts, fantasies or urges or be markedly distressed by them. Additional Features: Some individuals with Pedophilic Disorder are attracted only to males, others only to females, and others to both. Some individuals act on their pedophilic urges only with family members, while others have victims outside their immediate family or both.
Fetishistic Disorder	A) Over a period of at least 6 months, recurrent and intense sexual arousal from either the use of nonliving objects or a highly specific focus on nongenital body part(s), as manifested by fantasies, urges, or behaviors. B) The fantasies, sexual urges, or behaviors cause clinically significant distress or impairment in social, occupational, or other important areas of functioning. C) The fetish objects are not limited to articles of clothing used in cross-dressing (as in transvestic disorder) or devices specifically designed for the purpose of tactile genital stimulation (e.g., vibrator).	
Transvestic Disorder	A) Over a period of at least 6 months, recurrent and intense sexual arousal from cross-dressing, as manifested by fantasies, urges, or behaviors. B) The fantasies, sexual urges, or behaviors cause clinically significant distress or impairment in social, occupational, or other important areas of functioning.	
Other Paraphilic Disorder Involving Non-Consenting Individuals		A) A sustained, focused and intense pattern of atypical sexual arousal, as manifested by sexual thoughts, fantasies, urges, and/or behaviors, in which the focus of the arousal pattern involves others whose age or status renders them unwilling or unable to consent that is not specifically described in any of the other named Paraphilic Disorders categories (e.g., arousal patterns involving corpses or animals). B) The individual must have acted on these thoughts, fantasies or urges or be markedly distressed by them. C) The presentation does not satisfy the diagnostic requirements of Coercive sexual sadism disorder, Pedophilic disorder, Voyeuristic disorder, Exhibitionistic disorder, or Frotteuristic disorder.
Paraphilic Disorder Involving Solitary Behavior or Consenting Individuals		A) A sustained, focused and intense pattern of atypical sexual arousal, as manifested by sexual thoughts, fantasies, urges, and/or behaviors that involves consenting adults or solitary behavior. B) One of the following two elements must be present: 1.) The person is markedly distressed by the nature of the arousal pattern and the distress is not simply a consequence of rejection or feared rejection of the arousal pattern by others; or 2.) The nature of the paraphilic behavior involves significant risk of injury or death either to the individual (e.g., asphyxophilia or achieving sexual arousal by restriction of breathing) or to the partner (e.g., consensual sadism that results in injuries requiring medical treatment).
Otherwise Specified Paraphilic Disorder	A) Over a period of at least 6 months, recurrent and intense sexual arousal from a certain paraphilia that is not included other diagnoses, as manifested by fantasies, urges, or behaviors. B) The fantasies, sexual urges, or behaviors cause clinically significant distress or impairment in social, occupational, or other important areas of functioning. This diagnosis is applied when the paraphilia is not prevalent enough to include its own diagnosis; examples include telephone scatologia (obscene phone calls), necrophilia (sexual activity with corpses), zoophilia, coprophilia (being aroused by being defecated upon or defecating on others), and urophilia (being aroused by being urinated upon or urinating on others).	
Unspecified Paraphilic Disorder	A) Over a period of at least 6 months, recurrent and intense sexual arousal from an unspecified paraphilic disorder, as manifested by fantasies, urges, or behaviors. B) The fantasies, sexual urges, or behaviors cause clinically significant distress or impairment in social, occupational, or other important areas of functioning. This diagnosis is applied when the paraphilic disorder does not meet the full criteria for any of the other paraphilic disorders. It is used when a reason is not given for a specific paraphilic disorder criteria being specified because sufficient information may not be available.	A) A sustained, focused, and intense pattern of sexual arousal, as manifested by persistent unspecified paraphilic thoughts, fantasies, urges, or behaviors. B) The individual has acted on these thoughts, fantasies or urges or be markedly distressed by them. This category is an ‘unspecified’ residual category.

According to [28].

### Critical aspects of paraphilia diagnoses

Some experts in the field claim that it is unreasonable that pedophilia is the only paraphilic disorder that does not include specifiers for remission in the DSM-5 (e.g., [29]). In fact, there is no scientific evidence that pedophilic disorder is more intractable than any other disorder [20]. On the contrary, recent theories as well as empirical results indicate that sexual interest in children may change depending on the individuals’ motivation to change and their self-beliefs on being able to change their pedophilic interest [30–32]. Furthermore, patients report changes of their sexual interest in children as well as fixed pedophilic interests across the lifespan [29].

Another critical aspect concerns the fact that pedophilic disorder is still the only diagnosis for people who are sexually attracted to children. By definition, pedophilia refers to a sexual interest in prepubescent children [27]. Today, prepubescent children are aged 11 years or younger. A sexual interest in older children, i.e., pubescent children, aged approximately 11 to 14, is called hebephilia [33]. However, hebephilia is not included as a diagnosis in DSM-5 [27]. Thus, there is no appropriate diagnosis for people who are sexually attracted to pubescent children. Researchers state that this leads to substantial problems both in clinical practice and in research as professionals are not able to differentiate sufficiently between pedophilic and hebephilic individuals [20].

Finally, few experts fundamentally question why medicine even pathologizes sexual interests by including paraphilia diagnoses in diagnostic manuals like the DSM and the ICD (e.g., 4, 12, 34]. They argue that no data basis justifies the previous and current dealing with individuals who have paraphilic interests. The diagnosis of a paraphilic disorder promotes stigmatization and causes individuals to experience bias in healthcare, psychotherapy, the workplace, as well as political and social spheres [34, 35]. In addition, they state that current diagnostic criteria pose the danger of abuse in forensic contexts. People who have committed a sexual offense and are placed in psychiatric institutions because of a paraphilia diagnosis can continue to be unjustly imprisoned based on the diagnosis - even if they have long since served their prison sentence [4]. In contrast, there is the question on what basis practitioners could help people who, for example, suffer from their sexual interest in children and/or are at risk of committing sexual assaults on children if there was no diagnosis for their condition.

Due to the ongoing disagreement between experts, the debate on paraphilias will probably continue in the future. However, the fact that paraphilic interests play a role in clinical practice ever raises the question of how to treat individuals who suffer from their sexual interests or who commit sexual offenses related to paraphilias. In the following, a short description of the development of treatment approaches for paraphilic disorders is provided.

### Development of treatment approaches for paraphilic disorders and individuals who commit sexual offenses

During the late 19th century, when paraphilias were considered to be medical phenomena, the first treatment approach was surgical castration. It was first applied in 1892 in Switzerland for a patient who suffered from hypersexuality [36]. During the 1940s, clinicians began to use medications to treat those who had committed sexual offenses. The first attempts of medications have been considered unethical [37], such as surgical castration [38]. More recent attempts in medical treatment approaches include the use of luteinizing hormone-releasing hormone (LHRH) agonists which reduce testosterone to very low levels and are associated with very low levels of recidivism (e.g., [39–46]. However, LHRH agonists come along with problematic side effects, i.e., osteoporosis and reduction in bone mineral density [47]. This is why practitioners are recommended to evaluate costs and benefits and to cooperate with endocrinologists and osteologists before prescribing testosterone-lowering medication [48]. In cases of low risk of recidivism, selective serotonin reuptake inhibitors (SSRIs) are used off-label as they decrease individuals’ sex drive [49].

Besides the medical treatment of paraphilias, different psychotherapeutic approaches have been developed simultaneously. Although Freud’s early psychoanalytically theory of sexuality [10] has been refined subsequently, it still builds the foundation of the psychoanalytic understanding of sexual deviance (e.g., [50–53]). Nevertheless, due to a lack of research, it remains unclear whether the theoretical basis of psychoanalysis in the understanding of the paraphilias is valid or whether it can improve the treatment success of paraphilias [47]. Behavioral therapy approaches were used from the 1960s. Aversion therapy—which was also developed for homosexuality—was applied to treat individuals who had committed sexual crimes [54, 55]. This therapy uses classical conditioning to induce a conditioned anxiety response (electric shocks) to a deviant stimulus which would result in a reduction of the inappropriate interest. Due to ethical concerns, aversion therapy had been left behind during the 1980s [55]. Other behavioral therapy approaches which are primarily used in the context of prison-based treatment programs for individuals who committed sexual offenses are cognitive behavioral therapy approaches. They demonstrated a reduction in recidivism with small but stable effects [56–58], especially when they are based on the Risk-Need-Responsivity (RNR) model by Andrew and Bonta [59].

In recent times and along with the growing digitalization of everyday life, web-based interventions are increasingly being developed for individuals who have paraphilic interests, e.g., sexual interest in children [60]. One website that addresses individuals with a sexual interest in children is the self-guided web-based intervention “Troubled Desire”. Users can complete a self-assessment questionnaire via the website and get feedback on their sexual preferences and problematic sexual behavior. Afterward, they have access to the web-based intervention and are supported in seeking further help if needed (e.g., contacting a therapist). “Troubled Desire” is based on a cognitive-behavioral approach including psychoeducation and worksheets [61, 62]. Another offer for individuals with sexual interest in children is “Help Wanted” which uses interactive videos to help people cope with their sexual attraction to children [63]. Other web-based interventions are guided by a professional. The cognitive-behavioral web-based intervention “Prevent It” addresses individuals who consume online abuse material and focuses on the reduction of consumption [64]. The web-based intervention “@myTabu” is also cognitive-behavioral oriented and targets individuals who committed sex crimes against children and are under community supervision. The intervention focuses on reducing the risk of recidivism [65]. In both interventions, users receive individual feedback from a professional for exercises they work on during their participation. Currently, both “Prevent It” and “@myTabu” are still under evaluation [60].

## Conclusion

Constantly changing social and cultural conditions determine how sexual behavior is assessed in the field of sexual medicine. Today, society and experts deem it necessary to protect the rights of people with atypical non-harmful sexual interests and therefore contribute to the minimization of stigmatization and discrimination against them. Thus, to clearly distinguish between paraphilic behaviors that are harmful to individuals and consensual deviant sexual behavior is becoming more and more important. However, even though there have already been so many changes in terms of paraphilic diagnoses, there are still challenges addressed by several authors (e.g., [2, 66]). Most important, the safety of especially vulnerable population groups, such as children, needs to be guaranteed in todays and future societies. Therefore, a balance between protecting paraphilic individuals’ rights and protecting vulnerable groups should always be pursued.
